# Buffalo Academy of Medicine

**Published:** 1902-01

**Authors:** Irving P. Lyon

**Affiliations:** Secretary


### SOCIETY PROCEEDINGS.

### Buffalo Academy of Medicine.

*Section on Pathology, October 15, 1901.*

Reported by IRVING P. LYON, M. D., Secretary.

This Section held its regular monthly meeting at the Academy rooms, Buffalo Public Library Building, Tuesday, October 15, 1901, Dr. Grover W. Wende, chairman, presiding.

The following papers were presented: The present status of our knowledge concerning the innervation of the heart, by Frederick C. Busch; and, Vegetating dermatitis developing during the course of infantile eczema, by Herman K. DeGroat and Grover W. Wende.

The lesions of this condition consist of pustulo-papules, upon which as a foundation a benign hypertrophy rapidly develops, resulting in a papillomatous, vegetating growth. They also reviewed other similar cases found in literature.

These papers were discussed by Drs. Williams, Van Peyma, Benedict and Lyon.

Members present, 26.

*Section on Pathology, November 19, 1901.*

REPORTED BY IRVING P. LYON, M. D., Secretary.

Tuesday, November 19, 1901, at 8.30 p.m., Dr. GROVER W. WENDE, chairman, presiding.

The essayist was Dr. LEWELLYS F. BARKER, professor of anatomy, University of Chicago, whose subject was, The unveiling of the cell.

The paper was discussed by Drs. Allen A. Jones, Williams, Howe, Busch, Barrows, Blaauw, Clewes, Howell, Gaylord and Lyon.

Dr. Barker's paper will appear in a subsequent issue of the JOURNAL.

Members present, 58.

